# Flexible Two-Photon Interference Fringes with Thermal Light

**DOI:** 10.1038/s41598-017-02119-y

**Published:** 2017-05-16

**Authors:** De-Zhong Cao, Cheng Ren, Jin-Yang Ni, Yan Zhang, Su-Heng Zhang, Kaige Wang

**Affiliations:** 1 Department of Physics, Yantai University, Yantai, 264005 Shandong Province China; 2 College of Physics Science & Technology, Hebei University, Baoding, 071002 Hebei Province China; 3 Department of Physics, Applied Optics Beijing Area Major Laboratory, Beijing Normal University, Beijing, 100875 China

## Abstract

Flexible interference patterning is an important tool for adaptable measurement precisions. We report on experimental results of controllable two-photon interference fringes with thermal light in an incoherent rotational shearing interferometer. The two incoherent beams in the interferometer are orthogonally polarized, and their wavefront distributions differ only in an angle of rotation. The spacings and directions of the two-photon interference fringes vary with the rotation angle, as illustrated in three cases of two-photon correlation measurements in experiment.

## Introduction

Multi-photon interference distinguishes itself by narrowed fringe spacings. In principle, the fringe spacing in *N*-photon interference experiment becomes 1/*N* of that in the single-photon case. Therefore multi-photon interference is helpful to greatly improve precisions in quantum lithography^1^ and superresolved imaging^2, 3^. The subwavelength interference fringes were first reported with entangled two-photon sources^4, 5^. Later investigations showed that similar subwavelength fringes can be obtained with thermal light sources^6–8^. To overcome the imperfection that the two detectors must be placed at opposite positions, auxiliary devices, lenses^9^ and right-angle mirrors^10^ were used to flip the wavefront distribution, and thus two-photon interference fringes with thermal light correlation, where two detectors were located at the same position, were created.

Flexible interference fringes are the signs of adaptable precisions in optical metrology. As in classical lithography, the flexible interference patterning becomes an important tool to fabricate optical devices of artificial meta-materials, such as photonic crystals^11–13^. In correlated interference experiments with thermal light, the multi-photon interference fringes can be spaced out with various gaps, from ordinary gaps to superresolved gaps. Some attempts were made to achieve flexible correlated interference fringes by changing the velocities of the two scanning detectors^14–16^. In contrast to the previous investigations, we report on experimental results of flexible two-photon interference fringes with thermal light which are independent of measuring apparatuses. Their directions and fringe spacings vary with the rotation angle between the two overlapped thermal light beams in an incoherent rotational shearing interferometer. Various interference patterns are obtained in three cases of two-photon correlation measurements.

## Results

**Figure 1:**
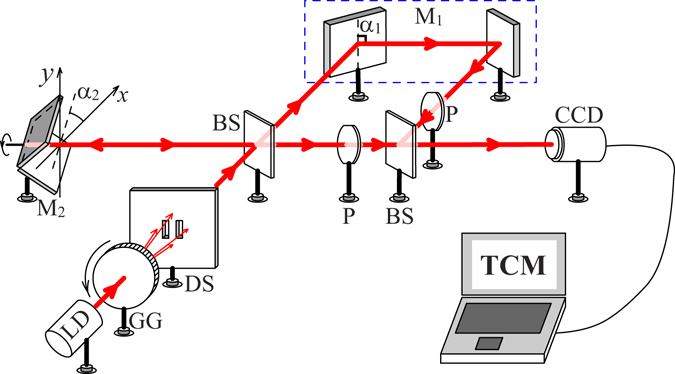
Experimental setup of wavefront rotation interferometer. LD is a laser diode of wavelength 650 nm. GG is a rotating ground glass disk. DS is composed of double slits. BS is the beam splitter. M_1_ and M_2_ are two right-angle mirrors, where M_1_ is composed of two separate sub-mirrors. P is the polarization plate. The two-photon correlation measurement (TCM) is implemented with a charge coupled device (CCD).

The experimental setup of flexible two-photon interference fringes with thermal light is shown in Fig. 1. A beam of pseudo-thermal light, which is obtained by projecting a laser beam (*λ* = 650 *nm*) onto a rotating ground glass (GG) disk, goes across two slits (DS: slit width *a* = 80 *μm*, height *b* = 680 *μm*, separation *d* = 310 *μm*) and then enters the incoherent rotational shearing interferometer. By a beam splitter BS in the interferometer, the thermal light beam is split into two daughter beams, which are then reflected back by two right-angle mirrors M_1_ and M_2_. For convenient adjusting, the right-angle mirror M_1_ is composed of two separate sub-mirrors, and its angle *α*_1_ = *π*/2 is fixed. The angle *α*_2_ of the right-angle mirror M_2_ varies freely. Two crossed polarizers (P) are used to ensure the two daughter beams orthogonal in the output plane. A charge coupled device (CCD) and a computer are used to perform two-photon correlation measurement (TCM) in the output port of the interferometer. The distance from the slits to the detection plane is *z* = 650 *mm*.

**Figure 2:**
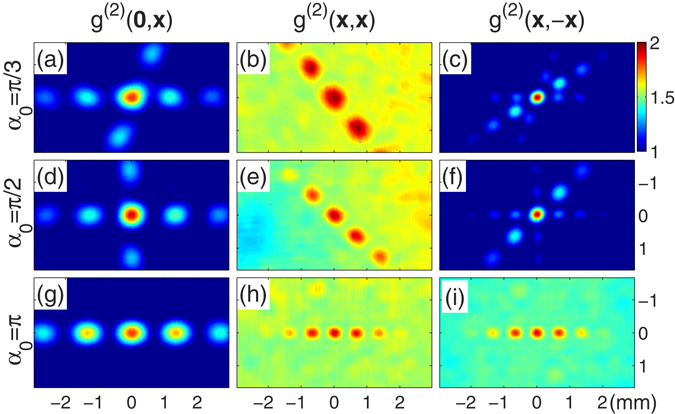
Experimental results of two-dimensional intensity correlation functions. In (**a**,**d**,**g**), ${g}^{\mathrm{(2)}}\mathrm{(0},\overrightarrow{x})$ is measured for *α*_0_ = *π*/3, *π*/2, and *π* respectively. In (**b**,**e**,**h**), ${g}^{\mathrm{(2)}}(\overrightarrow{x},\overrightarrow{x})$ is measured for *α*_0_ = *π*/3, *π*/2, and *π* respectively. In (**c**,**f**,**i**), ${g}^{\mathrm{(2)}}(\overrightarrow{x},-\,\overrightarrow{x})$ is measured for *α*_0_ = *π*/3, *π*/2, and *π* respectively. Each pattern is obtained by averaging 10,000 CCD frames.

As proved in the section of methods, the field in the detection plane is written as $E(\overrightarrow{x})={E}_{1}(\overrightarrow{x}){\hat{e}}_{1}+{E}_{2}(\overrightarrow{x}){\hat{e}}_{2}$, where *E*_1_ (*E*_2_) is the field from right-angle mirror M_1_ (M_2_), ${\hat{e}}_{1}$ and ${\hat{e}}_{2}$ are the orthogonal unit vectors ${\hat{e}}_{1}\cdot {\hat{e}}_{2}=0$. The two fields satisfy ${E}_{2}(\overrightarrow{x})={E}_{1}[\hat{R}(\,-\,{\alpha }_{0})\overrightarrow{x}]$, where $\hat{R}$ is the rotation matrix $$\hat{R}({a}_{0})=(\begin{array}{cc}\cos \,{\alpha }_{0} & -\sin \,{\alpha }_{0}\\ \sin \,{\alpha }_{0} & \cos \,{\alpha }_{0}\end{array})$$ with angle *α*_0_ = 2(*α*_2_ − *α*_1_). Figure 2 shows the experimental results of the measured two-photon (second-order) correlation functions ${g}^{\mathrm{(2)}}({\overrightarrow{x}}_{1},{\overrightarrow{x}}_{2})=\frac{\langle {E}^{\ast }({\overrightarrow{x}}_{1}){E}^{\ast }({\overrightarrow{x}}_{2})E({\overrightarrow{x}}_{2})E({\overrightarrow{x}}_{1})\rangle }{\langle {E}^{\ast }({\overrightarrow{x}}_{1})E({\overrightarrow{x}}_{1})\rangle \langle {E}^{\ast }({\overrightarrow{x}}_{2})E({\overrightarrow{x}}_{2})\rangle }$, each pattern is obtained by averaging over 10,000 CCD frames.

Figure 2(a,d,g) are the experimental results of two-photon correlation function ${g}^{\mathrm{(2)}}\mathrm{(0},\overrightarrow{x})$, where one detector is located at ${\overrightarrow{x}}_{1}=0$ while the other detector scans ${\overrightarrow{x}}_{2}=\overrightarrow{x}$, for rotation angles *α*_0_ = *π*/3, *π*/2, and *π*, respectively. Each pattern contains two groups of fringes, one is a set of normal interference fringes and the other is a set of rotated interference fringes with the angle *α*_0_. This confirms wavefront rotating of the incoherent interferometer. Both groups have approximately the same fringe spacings. The fringe visibility degrees are 0.2921 ± 0.0065, 0.3297 ± 0.0016, and 0.2843 ± 0.0009 for the patterns in Fig. 2(a,d,g), respectively. The two groups of fringes in Fig. 2(g) are completely overlapped because of the rotational angle *α*_0_ = *π*.

Figure 2(b,e,h) are the experimental results of two-photon correlation function ${g}^{\mathrm{(2)}}(\overrightarrow{x},\overrightarrow{x})$, where the two detectors move together ${\overrightarrow{x}}_{1}={\overrightarrow{x}}_{2}=\overrightarrow{x}$, for *α*_0_ = *π*/3, *π*/2, and *π*, respectively. There is only one group of fringes in each pattern, and these two-photon interference fringes vary with the rotation angle *α*_0_. We can see from Fig. 2(b,e,h) that the fringes are rotated by respective angles 2*π*/3, 3*π*/4, and *π*, and the fringe spacings are enlarged with respective enlargement ratios 0.8789 ± 0.0031, 0.6902 ± 0.0041, and 0.5029 ± 0.0043, compared with normal interference fringes. The two-photon interference fringes in Fig. 2(h), in fact, are the subwavelength interference fringes as in ref. 10. Moreover, the fringe visibility degrees are 0.1114 ± 0.0053, 0.1256 ± 0.0042, and 0.1270 ± 0.0013 in Fig. 2(b,e,h), respectively. By adjusting the rotational angle, we can therefore manipulate the fringe spacings and directions of the two-photon correlated patterns with thermal light.

Figure 2(c,f,i) are the experimental results of two-photon correlation function ${g}^{\mathrm{(2)}}(\overrightarrow{x},-\,\overrightarrow{x})$, where the two detectors scan symmetrically ${\overrightarrow{x}}_{1}=-\,{\overrightarrow{x}}_{2}=\overrightarrow{x}$, for *α*_0_ = *π*/3, *π*/2, and *π*, respectively. The fringe visibility degrees are 0.3373 ± 0.0002 and 0.3307 ± 0.0011 for the patterns in Fig. 2(c,f), both of which contains three groups of fringes. Two of them correspond to the ordinary and rotated subwavelength interference fringes (the rotational angle is *α*_0_). The third one is a set of enlarged interference fringes. In Fig. 2(i), however, there is only one group of fringe with visibility 0.1367 ± 0.0002. There should be three groups of fringes. In fact, owing to *α*_0_ = *π*, the ordinary and rotated subwavelength interference fringes are completely overlapped, while the enlargement ratio of the enlarged interference fringes tends to ∞ in Fig. 2(i). The infinitely enlarged fringes contribute an extra background and decreases the visibility. The enlargement ratios of the enlarged groups of fringes are 0.6012 ± 0.0023, 0.7086 ± 0.0021, and ∞ in Fig. 2(c,f,i), respectively.

According to the experimental results, we can obtain flexible two-photon correlated interference fringes, from the subwavelength fringes to enlarged fringes, by rotating the the right-angle mirror in the incoherent rotational shearing interferometer.

## Discussion

**Figure 3:**
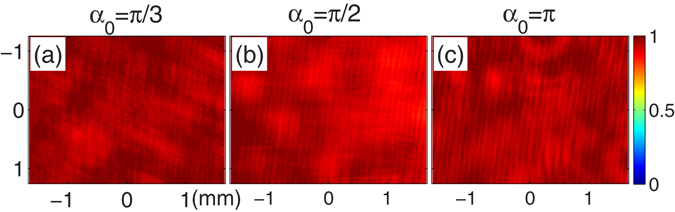
The experimental results of the normalized averaged intensity distributions for (**a**) *α*_0_ = *π*/3, (**b**) *α*_0_ = *π*/2, and (**c**) *α*_0_ = *π*.

A technique is established in the present paper to obtain flexible two-photon interference patterns of thermal light with the help of an incoherent rotational shearing interferometer. Two orthogonal polarizers were used to ensure the incoherence of the interferometer. Therefore the first-order interference does not exist in the intensity observation. That is, the averaged total intensity in the detection plane is uniform $\langle I(\overrightarrow{x})\rangle \propto \tfrac{4ab}{{\lambda }^{2}{z}^{2}}$. Figure 3 shows the experimental results of the normalized averaged intensity. Each experimental result is obtained by averaging over 10,000 CCD frames. The rotation angles are *α*_0_ = *π*/3, *π*/2, and *π* in Fig. 3(a–c), respectively. We can see that there are no any interference fringes, verifying the first-order incoherence of the source and the interferometer.

**Figure 4:**
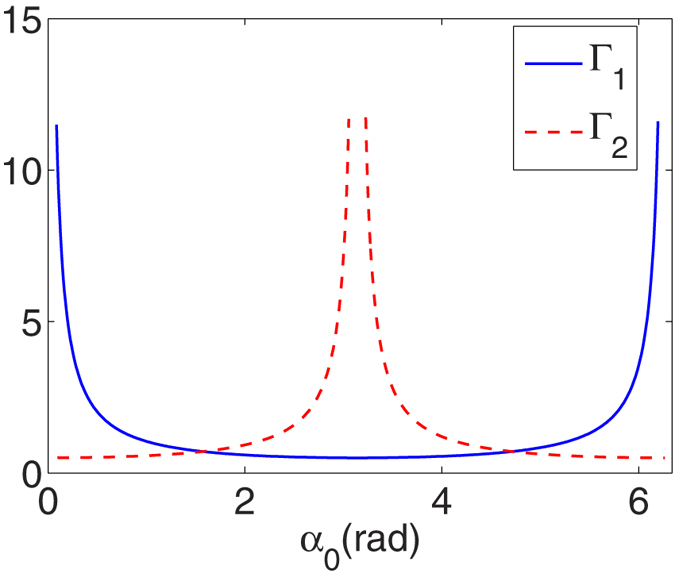
Enlargement ratios Γ_1_ and Γ_2_ versus the rotation angle *α*_0_. Γ_1_ and Γ_2_ are presented by the solid and dashed lines, respectively.

The interference information is retrieved in two-photon correlation measurements. We have retrieved various double-slit interference patterns by performing two-photon correlation measurements with thermal light. The fringe spacings and directions of the correlated interference patterns vary with the rotation angle. We define Γ_1_ as the enlargement ratios of the enlarged interference fringes in Fig. 2(b,e,h). We also define Γ_2_ as the enlargement ratios of the enlarged group of fringes in Fig. 2(c,f,i). The two ratios are plotted with solid and dashed lines, respectively, as shown in Fig. 4. Γ_1_ first decreases from ∞ to 1/2 and then increases from 1/2 to ∞ when the rotational angle *α*_0_ increases from 0 to 2*π*. The case is opposite for Γ_2_ that it first increases from 1/2 to ∞ and then decreases from ∞ to 1/2 when the rotational angle *α*_0_ increases from 0 to 2*π*.

The role of the two polarizers in the interferometer is to suppress possible single-photon interference, which can disturb two-photon interference. Figure 3 has verified that first-order interference does not exist at all. Besides, if the two slits are replaced by any other objects in experiment, what we can obtain in two-photon correlation measurements is the spatial spectrum of the object, as shown in Eq. (6). If there are no slits, however, we can obtain a two-photon spectrum of the source profile.

The rotational shearing interferometer had broad applications in conventional optics. Our present study on the incoherent rotational shearing interferometer will arouse new applications in incoherent pattern transformation with thermal light and quantum entangled light. In fact, the thermal light source in this experiment can be replaced by a two-photon entangled source, and similar two-dimensional interference patterns will be observed. This technique enriches multi-photon scenarios to obtain flexible interference fringes, and may be useful in optical metrology and correlated imaging.

## Methods

The field wavefront is symmetrically transformed with respect to the intersectional line of the right-angle mirror. The coordinate transformations of the two right-angle mirrors can be described by the two matrices

1 $${\hat{A}}_{j}=(\begin{array}{cc}\cos \,2{\alpha }_{j} & \sin \,2{\alpha }_{j}\\ \sin \,2{\alpha }_{j} & -\cos \,2{\alpha }_{j}\end{array}),$$

where *j* = 1, 2. It is obvious that ${\hat{A}}_{j}={\hat{A}}_{j}^{-1}$, ${\hat{A}}_{j}^{2}=1$, and

2 $${\hat{A}}_{2}=\hat{R}({\alpha }_{0}){\hat{A}}_{1}.$$

All the mirror reflectivities are assumed to be unity for convenient simplicity. So the fields reflected by the two right-angle mirrors have the relation ${E}_{2}(\overrightarrow{x})={E}_{1}[\hat{R}(\,-\,{\alpha }_{0})\overrightarrow{x}]$. This means that the right-angle mirrors can be used to set the rotation angle between the wavefronts of the two reflected fields, as shown in Fig. 1.

In the incoherent rotational shearing interferometer, the total detected field is $E(\overrightarrow{x})={E}_{1}(\overrightarrow{x}){\hat{e}}_{1}+{E}_{1}[\hat{R}(\,-\,{\alpha }_{0})\overrightarrow{x}]{\hat{e}}_{2}$, where the orthogonal polarizations are considered ${\hat{e}}_{1}\cdot {\hat{e}}_{2}=0$. The quantized fields in the detection plane can be written as

3 $${E}_{j}(\overrightarrow{x})\propto \int \tilde{D}({\overrightarrow{x}}_{s})\widehat{a}({\overrightarrow{x}}_{s})h({\overrightarrow{x}}_{s},{\hat{A}}_{j}\overrightarrow{x})d{\overrightarrow{x}}_{s},$$

where $\overrightarrow{x}$ and ${\overrightarrow{x}}_{s}$ are the transverse coordinates of the detected field and source field, respectively, $\widehat{a}({\overrightarrow{x}}_{s})$ is the photon annihilation operator in position presentation, $D(\overrightarrow{x})$ is the function of the source profile (two slits), the impulse response function has the form

4 $$h({\overrightarrow{x}}_{s},\overrightarrow{x})=\frac{1}{i\lambda z}{e}^{i\frac{2\pi z}{\lambda }+i\pi \frac{{|{\overrightarrow{x}}_{s}-\overrightarrow{x}|}^{2}}{\lambda z}}$$

in Fresnel diffraction, where *λ* is the wavelength, and *z* is the longitudinal distance.

By considering Eq. (3) and a simple state of thermal light^17^

5 $$\rho =\int {\widehat{a}}^{\dagger }({\overrightarrow{x}}_{s}){\widehat{a}}^{\dagger }({\overrightarrow{x}}_{s}^{^{\prime} })|0,0\rangle \langle 0,0|\widehat{a}({\overrightarrow{x}}_{s})\widehat{a}({\overrightarrow{x}}_{s}^{^{\prime} })d{\overrightarrow{x}}_{s}d{\overrightarrow{x}}_{s}^{^{\prime} },$$

where ${\widehat{a}}^{\dagger }({\overrightarrow{x}}_{s})$ is the photon creation operator and |0, 0〉 is the vacuum state, we calculate out the normalized second-order correlation function ${g}^{\mathrm{(2)}}({\overrightarrow{x}}_{1},{\overrightarrow{x}}_{2})$ as

6 $$\begin{array}{rcl}{g}^{\mathrm{(2)}}({\overrightarrow{x}}_{1},{\overrightarrow{x}}_{2}) & = & 1+\frac{1}{4}{|\tilde{D}({\overrightarrow{x}}_{2}-{\overrightarrow{x}}_{1})|}^{2}+\frac{1}{4}{|\tilde{D}[\hat{R}(-{\alpha }_{0}){\overrightarrow{x}}_{2}-{\overrightarrow{x}}_{1}]|}^{2}\\  &  & +\frac{1}{4}{|\tilde{D}[{\overrightarrow{x}}_{2}-\hat{R}(-{\alpha }_{0}){\overrightarrow{x}}_{1}]|}^{2}+\frac{1}{4}{|\tilde{D}[\hat{R}(-{\alpha }_{0})({\overrightarrow{x}}_{2}-{\overrightarrow{x}}_{1})]|}^{2},\end{array}$$

where $\tilde{D}(\overrightarrow{x})$ is the normalized Fourier transform of any source profile. For the two slits, it has

7 $$\tilde{D}(\overrightarrow{x})={\rm{sinc}}\,(\frac{\pi ax}{\lambda z})\,{\rm{sinc}}\,(\frac{\pi by}{\lambda z})\,\cos \,(\frac{\pi dx}{\lambda z}),$$

where *a* × *b* is the slit size, *d* is the slit separation, and sinc(*x*) = sin(*x*)/*x*. Equation (7) stands for the function of the field amplitude in the conventional Young’s two-slit interference experiment.

We now consider the first case that one detector is located at ${\overrightarrow{x}}_{1}=0$, while the other detector scans. The specific high-order correlation function is rewritten as

8 $${g}^{\mathrm{(2)}}\mathrm{(0,}\,\overrightarrow{x})=1+\frac{1}{2}{|\tilde{D}(\overrightarrow{x})|}^{2}+\frac{1}{2}{|\tilde{D}[\hat{R}(-{\alpha }_{0})\overrightarrow{x}]|}^{2}.$$

The first term in the right side of Eq. (8) is the uniform background. The second term contributes interference fringes as those in the conventional Young’s two-slit interference experiment. The third term contributes the rotated interference fringes with the angle *α*_0_. The fringe visibility degree, defined as $V=\frac{{g}_{{\rm{\max }}}^{\mathrm{(2)}}-{g}_{{\rm{\min }}}^{\mathrm{(2)}}}{{g}_{{\rm{\max }}}^{\mathrm{(2)}}+{g}_{{\rm{\min }}}^{\mathrm{(2)}}}$ with maximum ${g}_{{\rm{\max }}}^{\mathrm{(2)}}$ and minimum ${g}_{{\rm{\min }}}^{\mathrm{(2)}}$, can reach 1/3 for the whole pattern. These conclusions are illustrated by the experimental results in 2(a,d,j).

Next we consider the case that two detectors move together ${\overrightarrow{x}}_{1}={\overrightarrow{x}}_{2}=\overrightarrow{x}$. The normalized two-photon correlation function becomes

9 $${g}^{\mathrm{(2)}}(\overrightarrow{x},\overrightarrow{x})=\frac{3}{2}+\frac{1}{2}{|\tilde{D}[{\beta }_{1}\hat{R}(-\frac{{\alpha }_{0}+\pi }{2})\overrightarrow{x}]|}^{2},$$

where $\hat{R}(\frac{{\alpha }_{0}+\pi }{2})$ in Eq. (9) is the rotation matrix with angle (*α*_0_ + *π*)/2,

10 $${\beta }_{1}=2\,\sin \,\frac{{\alpha }_{0}}{2}.$$

We can see from Eq. (9) that the background is 3/2 and the fringe visibility of the whole pattern can reach 1/7. Compared with the fringes in the conventional Yang’s two-slit interference experiment, the fringes in Eq. (9) are enlarged with ratio Γ_1_ = 1/|*β*_1_|, and are rotated with angle (*α*_0_ + *π*)/2. The enlargement ratio satisfies Γ_1_ ≥ 1/2. These conclusions are illustrated by the experimental results in 2(b,e,h). Especially in the case of rotation angle *α*_0_ = *π*, subwavelength interference fringes can be obtained since *β*_1_ = 2 and Γ_1_ = 1/2, as shown in 2(h).

In the last case, the two detectors scan symmetrically ${\overrightarrow{x}}_{1}=-\,{\overrightarrow{x}}_{2}=\overrightarrow{x}$. The normalized intensity correlation function is

11 $${g}^{\mathrm{(2)}}(\overrightarrow{x},-\,\overrightarrow{x})=1+\frac{1}{4}{|\tilde{D}\mathrm{(2}\overrightarrow{x})|}^{2}+\frac{1}{4}{|\tilde{D}\mathrm{(2}\hat{R}(-{\alpha }_{0})\overrightarrow{x})|}^{2}+\frac{1}{2}{|\tilde{D}[{\beta }_{2}\hat{R}(-\frac{{\alpha }_{0}}{2})\overrightarrow{x}]|}^{2},$$

where

12 $${\beta }_{2}=2\,\cos \,\frac{{\alpha }_{0}}{2}.$$

The first term in Eq. (11) contributes background 1, and other three terms contribute three groups of interference fringes. The first group of fringes are the ordinary subwavelength interference fringes. The second group of fringes are the rotated subwavelength interference fringes with angle *α*_0_. The third group of fringes, compared with the normal Young’s interference fringes, are enlarged with ratio Γ_2_ = 1/|*β*_2_| and rotated with angle *α*_0_/2. The enlargement ratio satisfies Γ_2_ ≥ 1/2. These conclusions are illustrated by the experimental results in 2(c,f,i).

By the way, the enlargement ratios Γ_1_ and Γ_2_ meet $\frac{1}{{{\rm{\Gamma }}}_{1}^{2}}+\frac{1}{{{\rm{\Gamma }}}_{2}^{2}}=4$, and are plotted with solid and dashed lines, respectively, as shown in Fig. 4.
